# Improving cellular nuclei detection in histological images by combining super-resolution techniques and deep learning-based object detectors

**DOI:** 10.1007/s11517-026-03604-1

**Published:** 2026-08-13

**Authors:** Eduardo Díaz-Gaxiola, Ivan García-Aguilar, Arturo Yee-Rendón, Ines F. Vega-lópez, Ezequiel López-Rubio, Rafael Marcos Luque-Baena

**Affiliations:** 1 Facultad de Informática Culiacán, Universidad Autónoma de Sinaloa, Ciudad Universitara, Culiacán, 80040 Sinaloa México; 2 ITIS Software, Universidad de Málaga, C/Arquitecto Francisco Peñalosa 18, Málaga, 29010 Spain; 3 Biomedic Research Institute of Málaga, IBIMA Plataforma BIONAND, C/Doctor Miguel Díaz Recio, 28, Málaga, 29010 Spain

**Keywords:** Object detection, Convolutional neural networks, Super-resolution, Multiscale fusion, Digital histopathology

## Abstract

**Abstract:**

Digital pathology has enabled large-scale analysis of histological images. However, accurate detection of cellular nuclei remains challenging due to variability in morphology, staining, and especially image resolution. Existing object detection approaches often degrade when applied to low-resolution images, and current solutions typically address either multi-scale detection or image enhancement independently. In this work, we propose a hybrid framework that integrates super-resolution with a dual-branch detection strategy combining full-image and patch-based inference. This design leverages both global contextual information and localized high-detail analysis to improve detection robustness. The outputs of both branches are fused through a confidence-weighted mechanism followed by non-maximum suppression and clustering-based refinement. The proposed method was evaluated on the NuCLS dataset, demonstrating consistent improvements over baseline detection approaches. In particular, the combined workflow achieved up to a 20% increase in mAP@0.5–0.95 at higher confidence thresholds, achieving competitive performance compared to state-of-the-art methods while maintaining a lightweight architecture. These results highlight the effectiveness of integrating super-resolution and multi-scale detection strategies for improving nuclei detection in histopathological images.

**Graphical abstract:**

**Figure:**
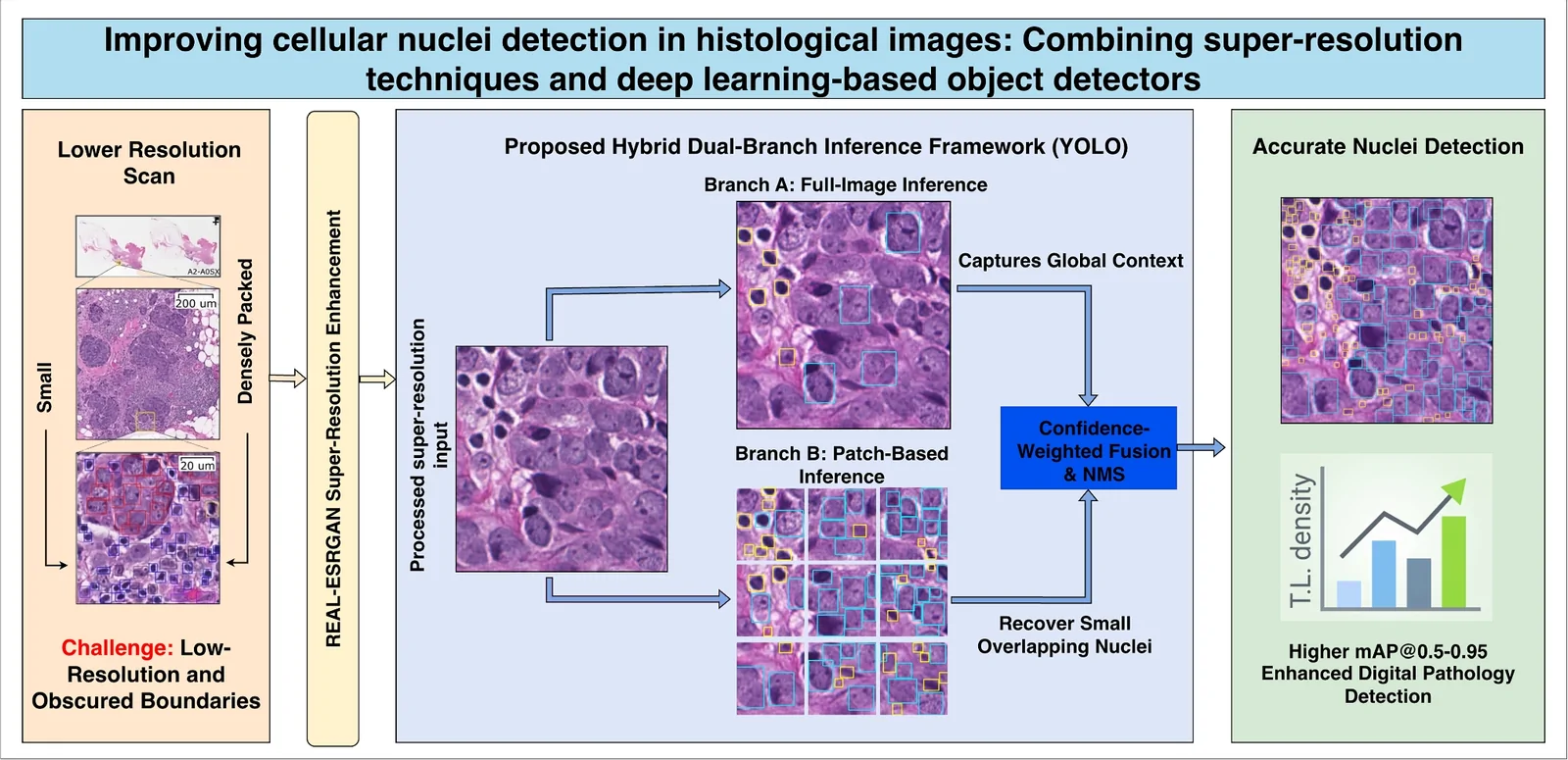

## Introduction

Accurate identification of cellular nuclei in histopathological imagery constitutes a fundamental step toward correct diagnosis and prognosis. Many computer-aided diagnostic systems, as well as tissue-grading protocols, first locate every nucleus within the specimen. In breast cancer, systems such as Nottingham evaluate the nuclear shape, size, and pleomorphism to estimate tumor aggressiveness [1, 2]. Moreover, the density of tumor-infiltrating lymphocytes (TIL) has been associated with overall survival in large meta-analyses [3] and in studies involving more than 3,700 patients treated with neoadjuvant chemotherapy [4]. In specific contexts, automatically generated nuclear metrics outweigh conventional biomarkers: a deep-learning algorithm that scores nuclear pleomorphism exhibited greater prognostic power than hormone status assessed by immunohistochemistry [5] and yielded consistent results in external cohorts [6].

Computer-aided diagnosis (CAD) systems have become increasingly important in medical imaging, assisting clinicians in the detection, characterization, and diagnosis of pathological conditions. These systems typically integrate multiple stages, including image preprocessing, feature extraction, detection, and classification, to support clinical decision-making. In recent years, deep learning-based approaches have been successfully applied across a wide range of domains, including medical imaging tasks such as anatomical landmark detection in colonoscopy and automated analysis in capsule endoscopy, as well as broader applications in artificial intelligence, such as model optimization, signal processing, and predictive modeling [7–12]. These works collectively demonstrate the versatility and effectiveness of deep learning in improving accuracy, robustness, and efficiency across complex tasks.

In the context of histopathology, accurate nucleus detection represents a critical initial step for downstream tasks such as grading and prognosis estimation. Recent work, such as the CAD framework proposed for brain tumor detection in MRI [13], demonstrates how combining advanced image processing with deep learning models can significantly improve diagnostic performance and reliability. Inspired by these developments, our work can be interpreted as a specialized CAD component focused on robust nucleus detection under challenging imaging conditions.

Within the fields of computer vision and deep learning, significant advances have driven progress in nucleus-level detection. Convolutional Neural Network (CNN)-based object detectors generate bounding boxes for individual nuclei, providing a direct and efficient solution for tasks such as counting and localization. One-stage models such as YOLOv3 [14] and EfficientDet [15] process multiple images per second with high accuracy, while two-stage architectures like Faster R-CNN [16] and its extension Mask R-CNN [17] continue to lead in instance segmentation benchmarks. Numerous studies have adapted these detectors to histopathology: for instance, HD-YOLO incorporates a BiFPN and a mask head for fine-grained segmentation, outperforming earlier methods in both precision and computational efficiency [18]. Enhancements to Mask R-CNN have further improved panoptic quality on publicly available histology datasets [19], while multi-scale contextual approaches help preserve sensitivity in the presence of heterogeneous cellular density patterns [20].

Despite their success, existing approaches exhibit several limitations. Many object detection models are highly sensitive to image resolution and often fail to detect small or densely packed nuclei when applied to downsampled images. Additionally, while multi-scale architectures improve detection performance, they do not explicitly address the loss of fine-grained details caused by resolution degradation. Conversely, super-resolution methods enhance image quality but are rarely integrated into detection pipelines in a unified manner. These limitations highlight the need for approaches that jointly address both resolution enhancement and multi-scale detection.

Nevertheless, several challenges must be addressed particularly maintaining accuracy when image resolution is sub-optimal. Slides are routinely scanned at 20× or 40× magnification, generating multi-gigabyte files that strain both storage and processing resources [21]. To reduce space requirements, many laboratories downsample images or delete older scans. Evidence shows that lowering resolution reduces sensitivity to small objects unless specialized multi-scale strategies are applied [22]. Super-resolution (SR) has emerged as a promising solution to this problem. Generative models such as SRGAN [23] and Restore-GAN, explicitly trained for digital pathology [24], can reconstruct lost nuclear detail. Systematic reviews confirm that deep learning techniques outperform traditional methods in medical image processing, and the recent HistoCell study demonstrated that inferring super-resolved cellular profiles from H&E-stained images can yield concrete clinical benefits [25].

Several recent studies in state-of-art have addressed nucleus detection and histopathology image analysis, each with differing focuses and methodological strategies. Diniz et al. [26] employed a two-stage ensemble of CNN classifiers and traditional post-processing to detect overlapping cervical nuclei, achieving high accuracy but relying heavily on handcrafted components. Yang et al.’s [27] ADS_UNet advanced segmentation performance through dense nested U-Net structures and attention mechanisms but remained limited to pixel-wise outputs without resolution-aware inference. Xie et al. [28] proposed DB-FCN, a dual-branch detection network with attention-based feature fusion, offering improvements in detection accuracy yet constrained to in-network fusion. Shi et al. [29] introduced a segmentation model enhanced by global context-aware modules and hierarchical feature aggregation, effective for boundary refinement but not tailored for object-level detection.

This work explores whether combining SR with advanced detectors can enhance nuclear localization, especially when images originate from lower-resolution scanners. A meta-model combining SR and detection is proposed and evaluated against SR-only and detection-only baselines using the NuCLS dataset [30], which includes over 220,000 annotated nuclei from breast carcinoma specimens. The diversity of NuCLS provides a rigorous testbed for detectors such as YOLO, EfficientDet, and Faster R-CNN, laying the foundation for robust clinical applications.

Despite significant advances in deep learning-based nucleus detection, existing approaches present notable limitations. Most methods either rely on multi-scale detection strategies without explicitly addressing image resolution degradation, or apply super-resolution techniques independently of the detection pipeline. Consequently, there is a lack of integrated frameworks that jointly exploit resolution enhancement and multi-scale contextual information for robust nucleus detection. To address these limitations, this work proposes a hybrid detection framework that combines super-resolution with a dual inference strategy. The main contributions of this study are summarized as follows: (i) the integration of super-resolution as a preprocessing step to enhance low-resolution histopathological images, (ii) a dual-branch detection approach combining full-image and patch-based inference to capture both global and local features, (iii) a confidence-weighted fusion strategy with clustering-based post-processing to improve detection consistency, and (iv) a comprehensive evaluation on the NuCLS dataset demonstrating improved performance over baseline and state-of-the-art methods.

The remainder of this article is organized as follows: Section 2 details the proposed methodology, including the SR scheme and the object detectors evaluated. Section 3 outlines the experimental design, introducing the NuCLS dataset along with the training and inference configurations. Section 4 presents and discusses the quantitative and qualitative results, comparing SR-only, detection-only, and the proposed meta-model. Finally, Section 5 offers the conclusions and outlines directions for future research.

## Methodology

**Fig. 1:**
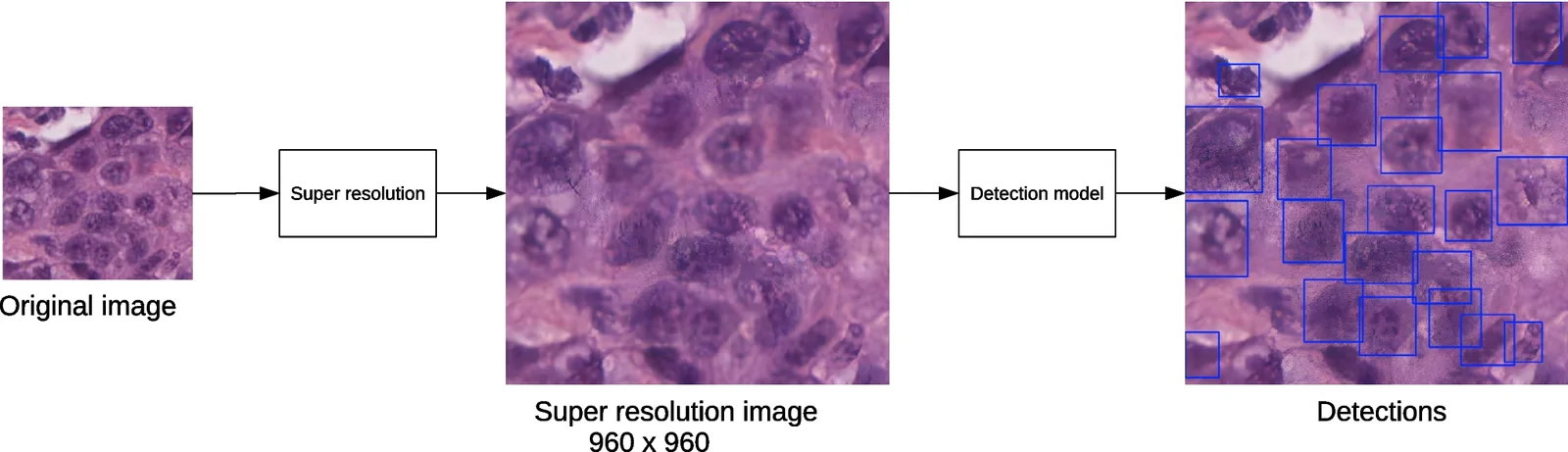
Full-image inference workflow, where super-resolution is applied to the entire image before detection

**Fig. 2:**
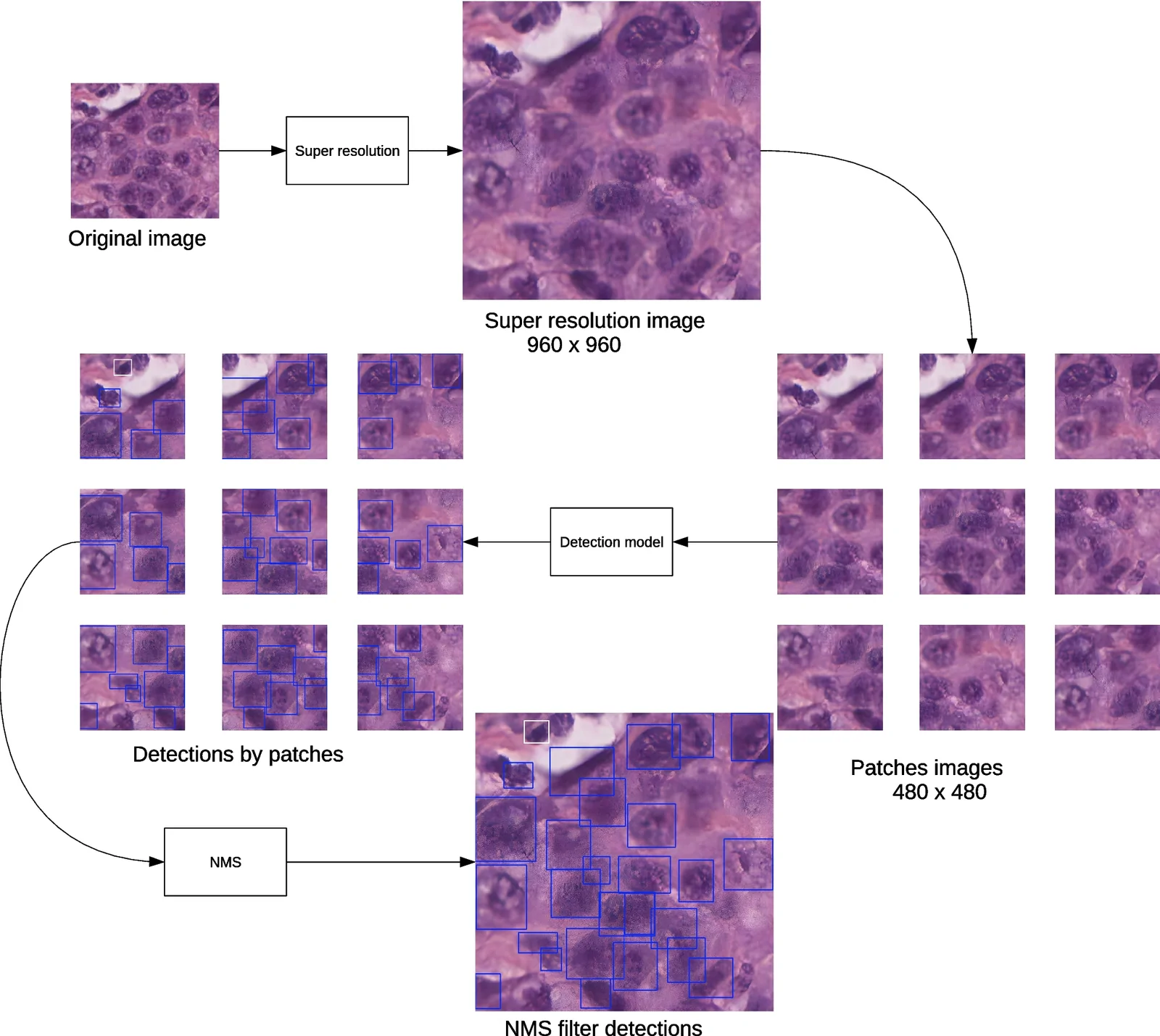
Patch-based inference workflow, involving division into patches and independent detection on each patch after super-resolution

**Fig. 3:**
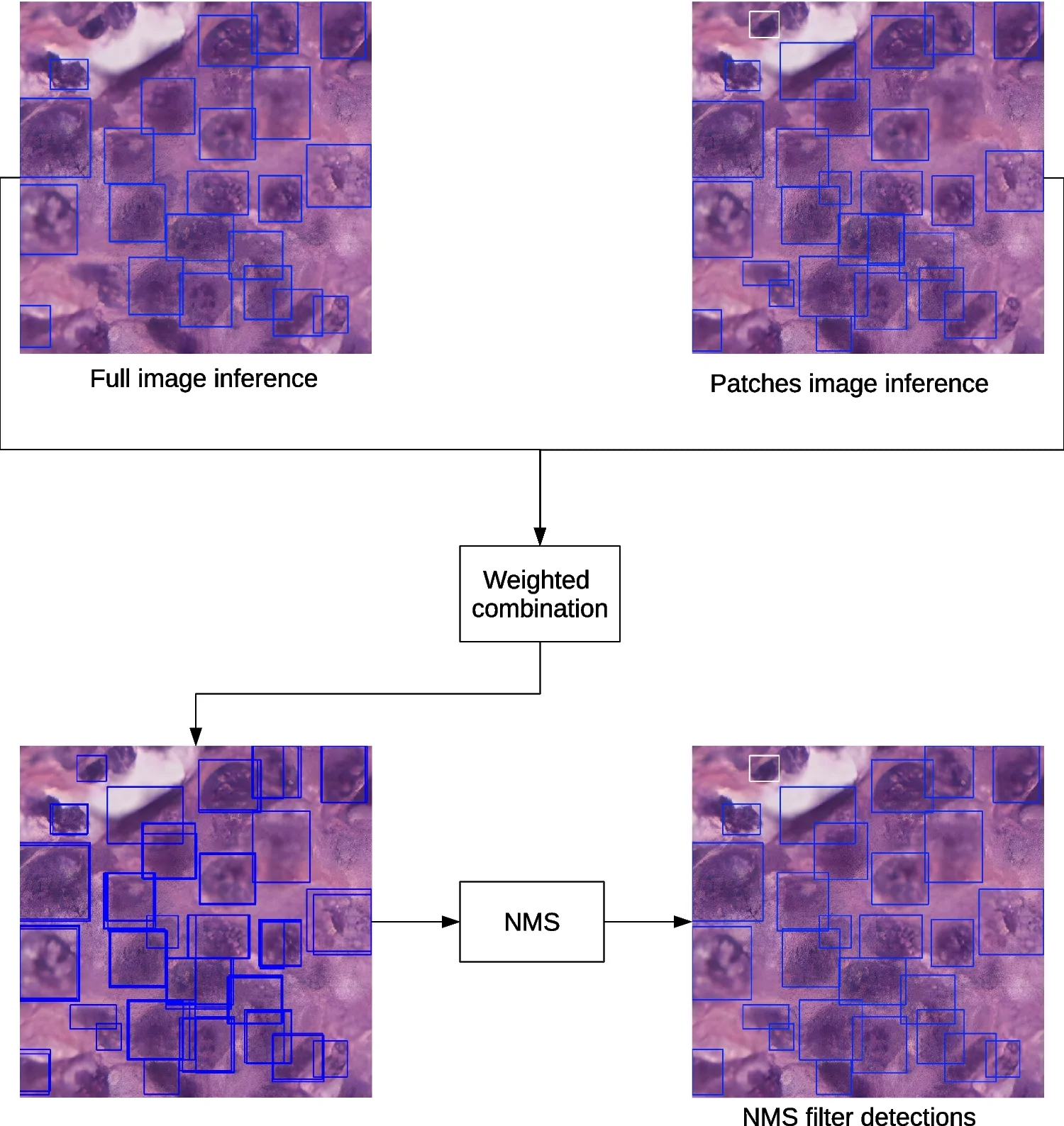
Fusion of full-image and patch-based detections through weighted confidence scores followed by non-maximum suppression

This section presents a comprehensive methodology for object detection that combines two complementary workflows: full-image inference (Fig. 1) and patch-based inference (Fig. 2). Both workflows utilize super-resolution preprocessing to enhance image quality, which improves the accuracy of subsequent detection. The detections from both workflows are then combined through a weighted fusion process and refined with post-processing steps that cluster overlapping bounding boxes and assign unified class labels and confidence scores (Fig. 3). Detailed explanations and formulas are provided for each step.

### Preprocessing and notation

The input to the system is a low-resolution RGB image $\textbf{X}_{LR} \in \mathbb {R}^{H \times W \times 3}$, where *H* and *W* are the image height and width, respectively. Since detection accuracy is typically degraded by low-resolution inputs, a super-resolution network $\mathcal {F}$ is applied to produce a high-resolution image $\textbf{X}_{HR}$ of fixed size *S × S* pixels, where *S* is chosen based on computational and model requirements (commonly *S=960*). This process is formally written as:

1 $$\begin{aligned} \textbf{X}_{HR} = \mathcal {F}(\textbf{X}_{LR}). \end{aligned}$$

The scaling factor *f* relates the dimensions of the original and super-resolved images:

2 $$\begin{aligned} f = \frac{S}{\max (H, W)}, \end{aligned}$$

meaning that the original image is scaled by a factor *f* in both dimensions to achieve the resolution *S × S*.

### Super-resolution

The super-resolution function $\mathcal {F}$ is implemented using Real-ESRGAN, a generative adversarial network (GAN)-based model designed for real-world image super-resolution. Real-ESRGAN extends the ESRGAN framework by incorporating a more realistic degradation modeling strategy and improved training stability, enabling it to recover fine-grained textures and structural details from low-quality inputs. In this work, a pre-trained Real-ESRGAN model is employed without additional fine-tuning on histopathology-specific data. This choice is motivated by its strong generalization capability and its effectiveness in restoring visually meaningful details, which are critical in nuclear pathology for preserving morphological features such as nuclear membranes, boundaries, and chromatin texture. The scaling factor is defined as in Eq. (2), where the input image is resized to a fixed resolution of *S × S*. The super-resolution model is applied as a preprocessing step prior to detection, ensuring that both the full-image and patch-based workflows operate on enhanced images with improved spatial resolution. Although the model is trained on generic natural images, its ability to reconstruct high-frequency details proves beneficial for histopathological analysis. Nevertheless, domain-specific fine-tuning could further improve the preservation of clinically relevant features and is considered a direction for future work.

Detections output by the model are represented as tuples:

3 $$\begin{aligned} \textbf{s}_i = (\alpha _i, \beta _i, \gamma _i, \delta _i, \epsilon _i, \omega _i), \quad i=1,\ldots ,N, \end{aligned}$$

where $(\alpha _i, \beta _i)$ and $(\gamma _i, \delta _i)$ denote the upper-left and lower-right coordinates of the bounding box within the image, $\epsilon _i$ is the class label chosen from a set $\mathcal {C}$ of possible classes, and $\omega _i \in [0,1]$ is the confidence score expressing the detection’s certainty.

### Workflow 1: Full-image inference

The first detection workflow (Fig. 1) operates on the entire super-resolved image $\textbf{X}_{HR}$. The detector $\mathcal {M}_{\text {full}}$ processes the image and produces a set $S_{\text {full}}$ of *N* detections:

4 $$\begin{aligned} S_{\text {full}} = \mathcal {M}_{\text {full}}(\textbf{X}_{HR}) = \left\{ \textbf{s}_i = (\alpha _i, \beta _i, \gamma _i, \delta _i, \epsilon _i, \omega _i) \mid i=1, \ldots , N \right\} . \end{aligned}$$

Due to potential multiple overlapping detections for the same object, Non-Maximum Suppression (NMS) is applied separately per class to eliminate redundant bounding boxes. The NMS uses an Intersection over Union (IoU) threshold $t_{NMS}$ (commonly set to 0.5), where overlapping boxes with lower confidence scores are suppressed in favor of higher confidence ones. The NMS filtering step is expressed as:

5 $$\begin{aligned} S_{\text {full}} \leftarrow \textrm{NMS}_{\textrm{class}}(S_{\text {full}}, t_{NMS}). \end{aligned}$$

This workflow benefits from a global view of the entire image, enabling the detection of larger or well-defined objects but may struggle to localize smaller or densely packed objects.

### Workflow 2: patch-based inference

To complement the full-image approach, the super-resolved image $\textbf{X}_{HR}$ is divided into a grid of non-overlapping patches of size *P × P*, where *P < S* (for example, *P=480*). These patches enable focused, localized detection, which is particularly advantageous for identifying small or overlapping objects that might be missed when analyzing the entire image at once (Fig. 2).

The patches are indexed by (*m*, *n*), where $m, n \in \{0, \ldots , \lfloor S/P \rfloor - 1\}$, and defined as:

6 $$\begin{aligned} \textbf{P}_{m,n} = \textbf{X}_{HR}[mP : mP + P, \quad nP : nP + P]. \end{aligned}$$

Each patch $\textbf{P}_{m,n}$ is processed independently by a patch detector $\mathcal {M}_{\text {patch}}$, producing local detections:

7 $$\begin{aligned} S_{m,n} = \mathcal {M}_{\text {patch}}(\textbf{P}_{m,n}) = \left\{ \tilde{\textbf{s}}_i = (\tilde{\alpha }_i, \tilde{\beta }_i, \tilde{\gamma }_i, \tilde{\delta }_i, \epsilon _i, \omega _i) \mid i=1, \ldots , I \right\} . \end{aligned}$$

Since the bounding box coordinates are relative to the patch, a coordinate transformation is applied to map them to the global coordinate system of $\textbf{X}_{HR}$ by offsetting each coordinate by the patch’s position in the image:

8 $$\begin{aligned} \begin{aligned} \alpha _i&= \tilde{\alpha }_i + nP, \\ \beta _i&= \tilde{\beta }_i + mP, \\ \gamma _i&= \tilde{\gamma }_i + nP, \\ \delta _i&= \tilde{\delta }_i + mP. \end{aligned} \end{aligned}$$

All patch detections from every patch are combined into a single set:

9 $$\begin{aligned} S_{\text {patch}} = \bigcup _{m,n} S_{m,n}. \end{aligned}$$

Similarly to the full-image workflow, class-wise NMS with threshold $t_{NMS}$ is applied to this combined set to suppress redundant overlapping boxes:

10 $$\begin{aligned} S_{\text {patch}} \leftarrow \textrm{NMS}_{\textrm{class}}(S_{\text {patch}}, t_{NMS}). \end{aligned}$$

The patch-based approach enhances detection sensitivity for smaller objects or objects in crowded scenes by focusing detection on smaller image regions.

### Fusion of workflows

The final step involves fusing the filtered detections from both workflows, taking advantage of their complementary strengths (Fig. 3). The sets are merged as:

11 $$\begin{aligned} \hat{S} = S_{\text {full}} \cup S_{\text {patch}}. \end{aligned}$$

Recognizing that the full-image workflow typically yields more precise detections due to global context, confidence scores of its detections are scaled by a factor $w_f > 1$ (commonly $w_f=1.3$) before fusion, while patch-based detections retain their original confidence scores:

12 $$\begin{aligned} \omega _i^{\textrm{final}} = {\left\{ \begin{array}{ll} w_f \times \omega _i, & \textbf{s}_i \in S_{\text {full}}, \\ 1 \times \omega _i, & \textbf{s}_i \in S_{\text {patch}}. \end{array}\right. } \end{aligned}$$

This weighting scheme biases the fusion to favor full-image detections when the same object is detected in both workflows, while still leveraging the patch-based detections to recover objects missed at full-image scale.

After weighting, a final class-wise NMS with threshold $t_{NMS}$ is applied to the fused set to produce a unified, non-redundant detection set ready for further refinement.

### Post-processing: clustering and final selection

Despite the application of NMS, some overlapping bounding boxes corresponding to the same object may remain, especially in complex scenes. To address this, detections in the fused set $\hat{S}$ are clustered based on their spatial overlap: any pair of bounding boxes $\textbf{s}_p$ and $\textbf{s}_q$ with Intersection over Union (IoU) exceeding a threshold $t_{CL}$ (e.g., 0.7) are grouped into the same cluster $\mathcal {C}_k$:

13 $$\begin{aligned} {\text {IoU}}(\textbf{s}_p, \textbf{s}_q) > t_{CL}, \quad \textbf{s}_p, \textbf{s}_q \in \hat{S}. \end{aligned}$$

Within each cluster $\mathcal {C}_k$, the bounding boxes are aggregated to derive a single representative detection. First, the average bounding box coordinates are computed as the mean of all coordinates in the cluster:

14 $$\begin{aligned} \textbf{b}^{\textrm{avg}}_k = \frac{1}{|\mathcal {C}_k|} \sum _{\textbf{s}_i \in \mathcal {C}_k} (\alpha _i, \beta _i, \gamma _i, \delta _i). \end{aligned}$$

Then, the Euclidean distance between each bounding box $\textbf{b}_i = (\alpha _i, \beta _i, \gamma _i, \delta _i)$ and the average box $\textbf{b}^{\textrm{avg}}_k$ is computed:

15 $$\begin{aligned} D(\textbf{b}^{\textrm{avg}}_k, \textbf{b}_i) = \sqrt{\sum _{j=1}^4 \left( b^{\textrm{avg}(j)}_k - b_i^{(j)}\right) ^2}. \end{aligned}$$

The bounding box closest to the average is selected as the most representative bounding box for the cluster:

16 $$\begin{aligned} i^{*} = \arg \min _{i \in \mathcal {C}_k} D(\textbf{b}^{\textrm{avg}}_k, \textbf{b}_i). \end{aligned}$$

This approach avoids biasing the final detection toward any particular member and accounts for small variations in box coordinates.

To determine the class label for the cluster, majority voting is applied across all detections in the cluster:

17 $$\begin{aligned} \epsilon _k^* = \arg \max _{\epsilon \in \mathcal {C}} \sum _{i \in \mathcal {C}_k} \mathbb {I}(\epsilon _i = \epsilon ), \end{aligned}$$

where $\mathbb {I}$ is the indicator function that counts the number of detections belonging to class *ε*.

Finally, the confidence score for the cluster is assigned as the maximum confidence among its members:

18 $$\begin{aligned} \omega _k^* = \max _{i \in \mathcal {C}_k} \omega _i. \end{aligned}$$

This post-processing ensures a robust, consolidated output that leverages all detections to produce the most accurate and reliable bounding boxes with associated class labels and confidence levels.

### Parameter selection

The main parameters of the proposed framework, including the Non-Maximum Suppression threshold ($t_{NMS}$, clustering threshold ($t_{CL}$), and fusion weighting factor ($w_f$), were selected empirically based on preliminary experiments conducted on a validation subset of the dataset. The value of $t_{NMS}$ = 0.5 was chosen as a standard trade-off between removing redundant detections and preserving true positives. The clustering threshold $t_{CL}$ = 0.7 was selected to ensure that only highly overlapping detections are grouped, reducing the risk of merging distinct objects. The weighting factor $w_f$ = 1.3 was introduced to slightly favor detections from the full-image workflow, as it provides better global context, while still allowing patch-based detections to contribute complementary information. These values were found to provide a good balance between precision and recall without introducing excessive computational overhead.

## Material and methods

This section describes the different components that compose the workflow of the experiments. First, the dataset used to train the various object detection models is presented. Then, several object detection models are trained and evaluated. Finally, the results obtained by comparing the different object detection models using the proposed methodology are shown.

### Dataset

NuCLS [30] (nucleus classification, localization, and segmentation) is a comprehensive dataset for histopathological image analysis, focusing on nuclei in whole-slide scans of hematoxylin and eosin (H&E) stained breast carcinoma tissues. It draws data from 18 institutions contributing to The Cancer Genome Atlas (TCGA), covering a diverse range of clinical samples. The dataset includes 128,000 nucleus annotations from 3,944 fields of view (FOVs) across 125 patients, making it a valuable resource for developing and evaluating machine learning models aimed at improving the detection, classification, and segmentation of cellular structures in breast cancer tissue. NuCLS is divided into single-rater and multi-rater subsets. The single-rater set, curated specifically for training models on detection, classification, and segmentation tasks, comprises 1,744 images and 59,485 nucleus annotations. Each image represents a distinct FOV, with precise labeling of various nucleus types, providing a strong foundation for building algorithms that generalize well across diverse histopathological data. Originally, the NuCLS dataset was organized into 12 raw classes to represent the different types of nuclei observed in the images. However, following the methodology established by Amgad et al., these 12 raw classes were consolidated into three broader super-classes to simplify the classification task and improve model efficiency. These super-classes are: *tumor*, which encompasses all nuclei associated with tumor cells; *nonTIL*, referring to stromal cells that are not tumor-infiltrating lymphocytes (TILs); *sTIL*, which denotes tumor-infiltrating lymphocytes, a key immune response feature in cancerous tissues. This reorganization of classes helps reduce the complexity of the problem, focusing on broader, clinically relevant categories that are critical for cancer diagnosis and prognosis.

### Object detection models

To evaluate and compare the performance of different object detection approaches, we trained three representative models: Faster R-CNN ResNet50 [16], EfficientDet D0 [15], and YOLO V8 [31, 32]. All models were trained with the same hyperparameters: 100 epochs, batch size of 16, SGD optimizer with a learning rate of 0.001, momentum of 0.9, and weight decay of 0.0005. This setup allowed a fair comparison and supported the validation of our ensemble strategy.

#### Faster R-CNN

Faster R-CNN is a two-stage detector that first generates region proposals using a Region Proposal Network (RPN) and then refines them through a second stage for classification and bounding box regression. It leverages shared convolutional features to improve speed and efficiency. Anchors of multiple scales and aspect ratios are used to detect objects of varying sizes, and proposals are refined relative to these anchor boxes.

#### EfficientDet

EfficientDet is a lightweight yet powerful detector based on the EfficientNet backbone. It uses a Bi-directional Feature Pyramid Network (BiFPN) to fuse multi-scale features efficiently. The model applies compound scaling to jointly scale the backbone, feature network, and detection head, achieving a balance between accuracy and computational cost. EfficientDet-D0, the smallest model in the series, was selected for its efficiency in training and inference.

#### YOLO

YOLO (You Only Look Once) is a single-stage object detector that divides the input image into a grid and directly predicts bounding boxes and class probabilities for each cell. YOLO v8 enhances previous versions with architectural improvements for better performance and generalization. The model uses the Intersection over Union (IoU) metric for loss computation and applies Non-Maximum Suppression (NMS) to eliminate redundant detections.

## Results

In this section, we present a comparative analysis of the object detection models trained in our study. The evaluation focuses on three different model configurations: (i) a model trained on full-resolution images (*complete workflow*), (ii) a model trained on image patches (*patches workflow*), and (iii) a combined model that integrates detections from both approaches (*combine workflow*). To assess performance under varying detection priorities, we also explore how these models behave at different confidence score thresholds. This analysis highlights trade-offs between precision and recall and provides insights into the robustness of each method across different detection conditions.

### Evaluation metrics

Mean Average Precision (mAP) was employed as the primary evaluation metric, given its widespread acceptance in state-of-the-art object detection research as a standardized and reliable measure of model performance. This metric, which ranges from 0 to 1 values, captures both precision and recall across varying confidence thresholds, making it particularly suitable for a comprehensive evaluation of object detection tasks. In this study, mAP was reported under two configurations. The first, mAP@0.5, corresponds to an Intersection over Union (IoU) threshold of 0.5, whereby a predicted bounding box is considered correct if it overlaps the ground truth by at least 50%. This setting provides an initial assessment of performance under moderate localization requirements. The second configuration, mAP@0.5:0.95, reflects the average mAP computed over multiple IoU thresholds, ranging from 0.5 to 0.95 in increments of 0.05. This more rigorous criterion enables the evaluation of a model’s ability to localize objects with greater precision.

**Table 1:** Object detection performance across different confidence thresholds and workflows. Results are reported as mean ± standard deviation for both mAP@0.5 and mAP@0.5–0.95. The best results for each metric are highlighted in bold

		*Complete workflow*		*Patches workflow*		*Combine workflow*	
Model	Th	mAP50	mAP50–95	mAP50	mAP50–95	mAP50	mAP50–95
Faster-RCNN	0.3	*0.604 ± 0.19*	*0.312 ± 0.12*	*0.574 ± 0.19*	*0.296 ± 0.11*	$\mathbf {0.618} \pm \mathbf {0.19}$	*0.325 ± 0.12*
Faster-RCNN	0.4	*0.592 ± 0.20*	*0.308 ± 0.13*	*0.570 ± 0.19*	*0.295 ± 0.11*	*0.615 ± 0.19*	*0.325 ± 0.12*
Faster-RCNN	0.5	*0.569 ± 0.20*	*0.298 ± 0.12*	*0.566 ± 0.19*	*0.293 ± 0.11*	*0.611 ± 0.19*	*0.324 ± 0.12*
Faster-RCNN	0.6	*0.523 ± 0.20*	*0.277 ± 0.13*	*0.543 ± 0.18*	*0.285 ± 0.11*	*0.602 ± 0.19*	*0.320 ± 0.12*
EfficientDet	0.3	*0.531 ± 0.20*	*0.262 ± 0.11*	*0.564 ± 0.20*	*0.253 ± 0.11*	*0.583 ± 0.20*	*0.293 ± 0.12*
EfficientDet	0.4	*0.480 ± 0.20*	*0.239 ± 0.11*	*0.538 ± 0.20*	*0.243 ± 0.11*	*0.572 ± 0.20*	*0.290 ± 0.12*
EfficientDet	0.5	*0.393 ± 0.19*	*0.202 ± 0.11*	*0.463 ± 0.20*	*0.214 ± 0.11*	*0.541 ± 0.21*	*0.277 ± 0.12*
EfficientDet	0.6	*0.281 ± 0.18*	*0.150 ± 0.11*	*0.329 ± 0.20*	*0.175 ± 0.11*	*0.476 ± 0.21*	*0.247 ± 0.12*
YOLO V8 N	0.3	*0.559 ± 0.22*	*0.316 ± 0.14*	*0.524 ± 0.20*	*0.290 ± 0.13*	*0.588 ± 0.21*	*0.328 ± 0.13*
YOLO V8 N	0.4	*0.496 ± 0.23*	*0.285 ± 0.14*	*0.467 ± 0.20*	*0.266 ± 0.13*	*0.536 ± 0.21*	*0.304 ± 0.14*
YOLO V8 N	0.5	*0.417 ± 0.22*	*0.245 ± 0.14*	*0.406 ± 0.20*	*0.236 ± 0.13*	*0.476 ± 0.22*	*0.276 ± 0.14*
YOLO V8 N	0.6	*0.319 ± 0.20*	*0.193 ± 0.13*	*0.310 ± 0.20*	*0.182 ± 0.13*	*0.385 ± 0.21*	*0.227 ± 0.14*
YOLO V8 S	0.3	*0.567 ± 0.20*	*0.325 ± 0.14*	*0.557 ± 0.20*	*0.309 ± 0.13*	*0.597 ± 0.21*	$\mathbf {0.340} \pm \mathbf {0.14}$
YOLO V8 S	0.4	*0.510 ± 0.21*	*0.296 ± 0.14*	*0.523 ± 0.21*	*0.293 ± 0.13*	*0.570 ± 0.21*	*0.326 ± 0.14*
YOLO V8 S	0.5	*0.451 ± 0.21*	*0.267 ± 0.14*	*0.473 ± 0.22*	*0.268 ± 0.14*	*0.525 ± 0.21*	*0.304 ± 0.14*
YOLO V8 S	0.6	*0.360 ± 0.19*	*0.217 ± 0.13*	*0.400 ± 0.21*	*0.229 ± 0.13*	*0.452 ± 0.21*	*0.264 ± 0.13*
YOLO V8 M	0.3	*0.558 ± 0.21*	*0.323 ± 0.14*	*0.536 ± 0.21*	*0.303 ± 0.14*	*0.587 ± 0.21*	*0.336 ± 0.14*
YOLO V8 M	0.4	*0.516 ± 0.21*	*0.303 ± 0.14*	*0.495 ± 0.21*	*0.284 ± 0.14*	*0.558 ± 0.21*	*0.323 ± 0.14*
YOLO V8 M	0.5	*0.454 ± 0.21*	*0.271 ± 0.14*	*0.433 ± 0.21*	*0.252 ± 0.13*	*0.507 ± 0.21*	*0.298 ± 0.14*
YOLO V8 M	0.6	*0.379 ± 0.20*	*0.234 ± 0.13*	*0.346 ± 0.21*	*0.206 ± 0.13*	*0.428 ± 0.21*	*0.259 ± 0.14*
YOLO V8 L	0.3	*0.572 ± 0.21*	*0.323 ± 0.14*	*0.532 ± 0.20*	*0.298 ± 0.13*	*0.586 ± 0.21*	*0.333 ± 0.14*
YOLO V8 L	0.4	*0.530 ± 0.21*	*0.303 ± 0.14*	*0.495 ± 0.20*	*0.280 ± 0.13*	*0.564 ± 0.21*	*0.323 ± 0.14*
YOLO V8 L	0.5	*0.465 ± 0.21*	*0.272 ± 0.14*	*0.437 ± 0.20*	*0.252 ± 0.12*	*0.515 ± 0.21*	*0.298 ± 0.14*
YOLO V8 L	0.6	*0.390 ± 0.21*	*0.233 ± 0.14*	*0.360 ± 0.19*	*0.212 ± 0.12*	*0.447 ± 0.21*	*0.263 ± 0.13*
YOLO V8 X	0.3	*0.571 ± 0.20*	*0.322 ± 0.13*	*0.539 ± 0.21*	*0.298 ± 0.13*	*0.588 ± 0.20*	*0.332 ± 0.13*
YOLO V8 X	0.4	*0.524 ± 0.20*	*0.299 ± 0.13*	*0.509 ± 0.21*	*0.284 ± 0.13*	*0.566 ± 0.20*	*0.322 ± 0.13*
YOLO V8 X	0.5	*0.470 ± 0.20*	*0.272 ± 0.12*	*0.455 ± 0.22*	*0.259 ± 0.13*	*0.528 ± 0.20*	*0.305 ± 0.13*
YOLO V8 X	0.6	*0.406 ± 0.19*	*0.240 ± 0.12*	*0.379 ± 0.21*	*0.219 ± 0.12*	*0.469 ± 0.21*	*0.275 ± 0.13*

The quantitative results obtained from the evaluation of different object detection models under varying confidence thresholds and processing workflows are summarized in Table 1. This table presents the mAP@0.5 and mAP@0.5:0.95 values for each model across three distinct workflows: the complete workflow, the patches workflow, and the combined workflow. Confidence thresholds ranging from 0.3 to 0.6 were considered to explore the trade-off between detection sensitivity and precision.

From the results, it can be observed that the combined workflow generally led to improved performance compared to both the complete and patches workflows, particularly at higher confidence thresholds. While the gains were marginal at thresholds of 0.3 and 0.4, a more substantial improvement was noted at thresholds of 0.5 and 0.6. For instance, when using EfficientDet at a confidence threshold of 0.6, the complete workflow achieved 0.281 mAP@0.5 and 0.150 mAP@0.5:0.95, whereas the combined workflow attained 0.476 and 0.247, respectively. This corresponds to an absolute improvement of 0.195 in mAP@0.5 and 0.097 in mAP@0.5:0.95. Such improvements are attributed to the decreasing number of detections at higher thresholds, which affects both the complete and patches workflows individually. By merging detections from both strategies, the combined workflow compensates for missed instances and enhances localization precision. On average, this fusion approach resulted in an approximate 20% increase in mAP@0.5:0.95 at a confidence threshold of 0.6.

**Fig. 4:**
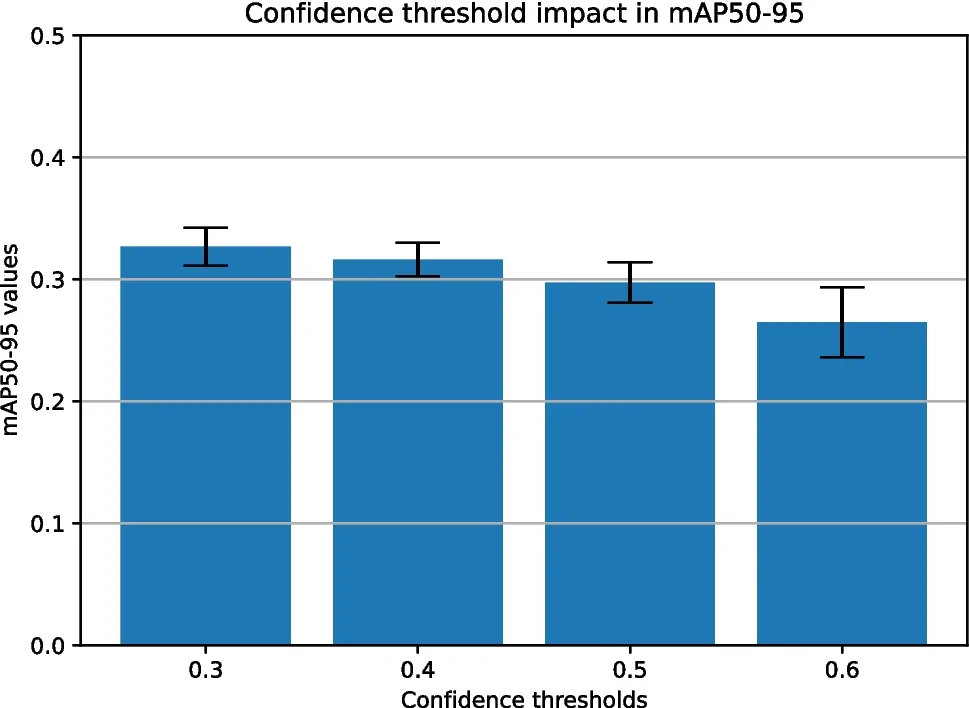
Impact of different confidence thresholds on mAP@0.5–0.95. Lower thresholds result in higher detection recall but may introduce more false positives, while higher thresholds improve precision at the cost of reduced recall

Figure 4 visually illustrates the impact of the confidence threshold on the mAP@0.50-0.95 metric. The confidence threshold achieving the best average results was 0.3, which corresponds to the most permissive setting. This average is computed across all the object detection models evaluated in the study. As the threshold value increases, the mAP tends to decrease. For instance, in the case of YOLO V8 S using the *complete workflow* (see Table 1), the mAP@0.50 decreased by 0.06 when the confidence threshold rose from 0.3 to 0.4.

**Fig. 5:**
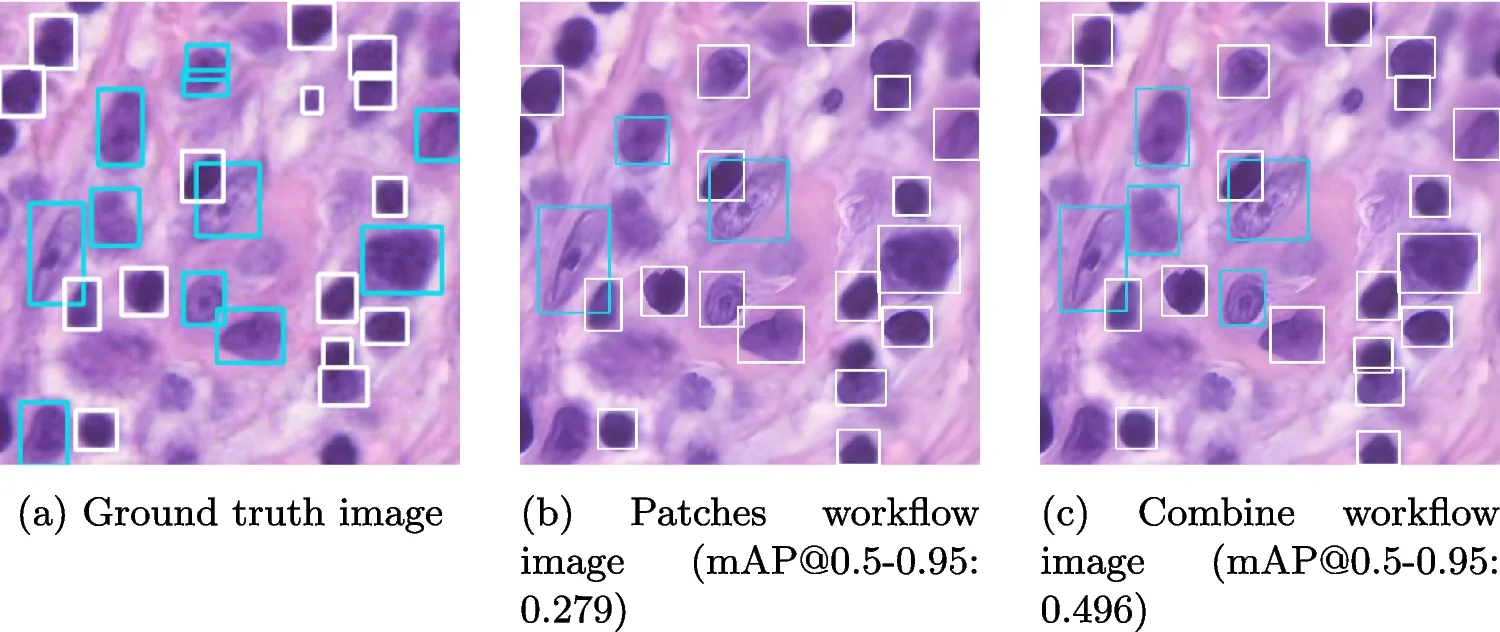
Visual comparison between ground truth annotations, patch-based workflow, and combined workflow at a confidence threshold of 0.5. The combined workflow produces detections that are more consistent with ground truth in both localization and bounding box size, leading to improved mAP scores

Figure 5 presents a visual comparison between ground truth (a), *patches workflow* (b) and *combined workflow* (c). The most notable differences between them two workflow strategies are the number of detections and their dimensions. Since the *combined workflow* selects the detections with the highest prediction values, they tend to be closer to the ground truth. This results in a higher mAP value at more restrictive IoU thresholds. This is demonstrated in the mAP@0.50-0.95 comparison, where the patches workflow achieves 0.279, while the combined workflow achieves 0.496.

To further validate the effectiveness of the proposed method, we compared our results with recent state-of-the-art approaches evaluated on the NuCLS dataset. In particular, the study by Amgad et al. [30], based on Mask R-CNN, represents one of the most relevant benchmarks for nucleus detection and classification. It is important to note that both approaches are evaluated under the same three-class configuration (tumor, nonTIL, sTIL), following the standard protocol derived from the NuCLS dataset. Our combined workflow based on YOLO V8-S achieved a slightly higher mAP@0.5–0.95 (0.340) compared to 0.330 reported by Amgad et al. In addition, our method exhibited lower variability, as reflected by the reduced standard deviation, indicating more stable performance across samples. However, it should be noted that the difference in mean performance is relatively small and should be interpreted with caution given the variability of the results. Therefore, rather than claiming a definitive improvement, the proposed method can be considered competitive with existing state-of-the-art approaches. It is also important to highlight that this performance is achieved with a significantly more efficient architecture. While the Mask R-CNN model used in [30] contains over 64 million parameters, our approach requires fewer than 10 million parameters. This represents a substantial reduction in computational complexity, making the proposed method more suitable for real-world deployment scenarios. Overall, the results demonstrate that the proposed integration of super-resolution and multi-scale detection provides a favorable trade-off between detection performance and computational efficiency.

After reviewing the state of the art, the study by Amgad et al. [30] emerged as the most representative work using the NuCLS dataset. Our *combined* workflow based on YOLO V8 S achieved the highest mAP@0.5–0.95, reaching **0.340**, and showed a markedly lower performance variability (standard deviation 0.14) than the Mask R-CNN model reported by Amgad et al. (mAP 0.330, std 0.90). For completeness, the *complete-slide* and *patch-based* YOLO pipelines tested here obtained mAP values of 0.325 and 0.309, respectively, with similarly low standard deviations (0.14 and 0.13). These results confirm the consistent advantage of the YOLO-based approach across all workflows.

A parameter-efficiency analysis reinforces this conclusion. YOLO V8 S delivered the best accuracy while using only 9.8 M parameters—roughly one-seventh of the 64 M parameters required by Mask R-CNN. Increasing the model size did not translate into higher accuracy: YOLO V8 M, L and X (23–62 M parameters) plateaued at mAP values between 0.336 and 0.332; Faster R-CNN (43 M) reached 0.325; and EfficientDet (3.8 M) lagged behind at 0.293. Even the ultra-light YOLO V8 N (2.7 M parameters) attained an mAP of 0.328, underscoring that our YOLO-based solution offers an optimal trade-off between computational cost and detection performance.

**Fig. 6:**
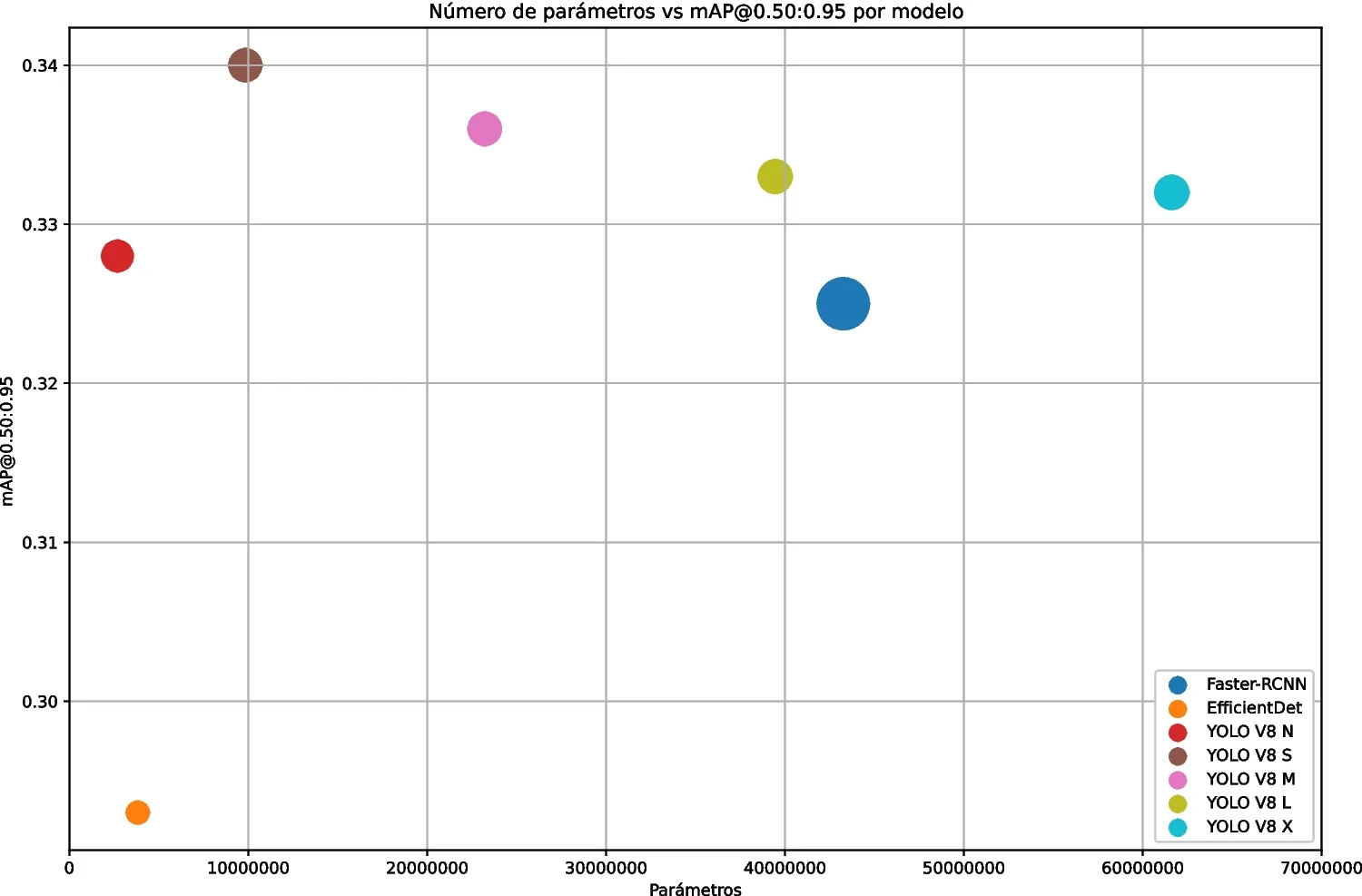
Relationship between model complexity (number of parameters) and detection performance (mAP@0.5–0.95). The size of each point represents inference time, highlighting the trade-off between accuracy, computational cost, and efficiency across different models

Figure 6 presents a visual comparison between the number of parameters and mAP@0.50-0.95. Additionally, the radius of each point on the plot represents the inference time, where a smaller radius indicates a shorter inference time, while a larger radius corresponds to a longer inference time.

**Fig. 7:**
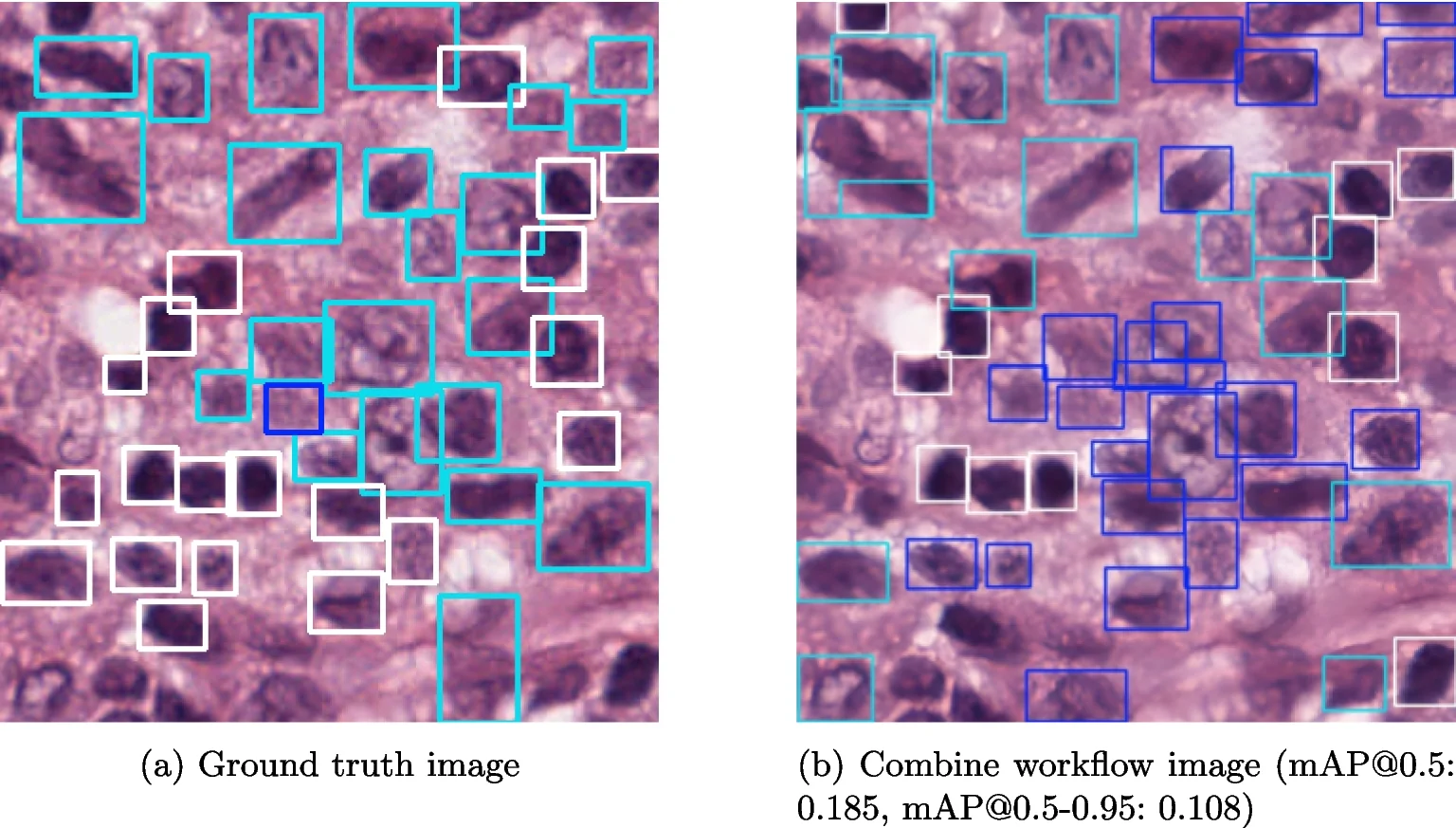
Example illustrating the classification challenge. Although the detected nuclei are spatially well localized, several instances are assigned incorrect class labels, which negatively impacts the overall mAP score. This highlights the difficulty in distinguishing visually similar classes such as nonTIL and sTIL

On the other hand, Fig. 7 highlights a recurring issue observed during the application of the detection models. This observation suggests that the primary limitation of the model is not localization accuracy but class discrimination. The high spatial alignment between predictions and ground truth indicates that the detection component is effective, while the classification branch remains the main source of error. When comparing the model outputs with the ground truth annotations, it was noted that, although the objects were spatially well localized, they were frequently assigned incorrect class labels. This misclassification negatively affected the mAP score. For example, in Fig. 7(b), the mAP@0.50-0.95 is just 0.108, despite a high visual and spatial correspondence between the predicted and actual objects.

**Fig. 8:**
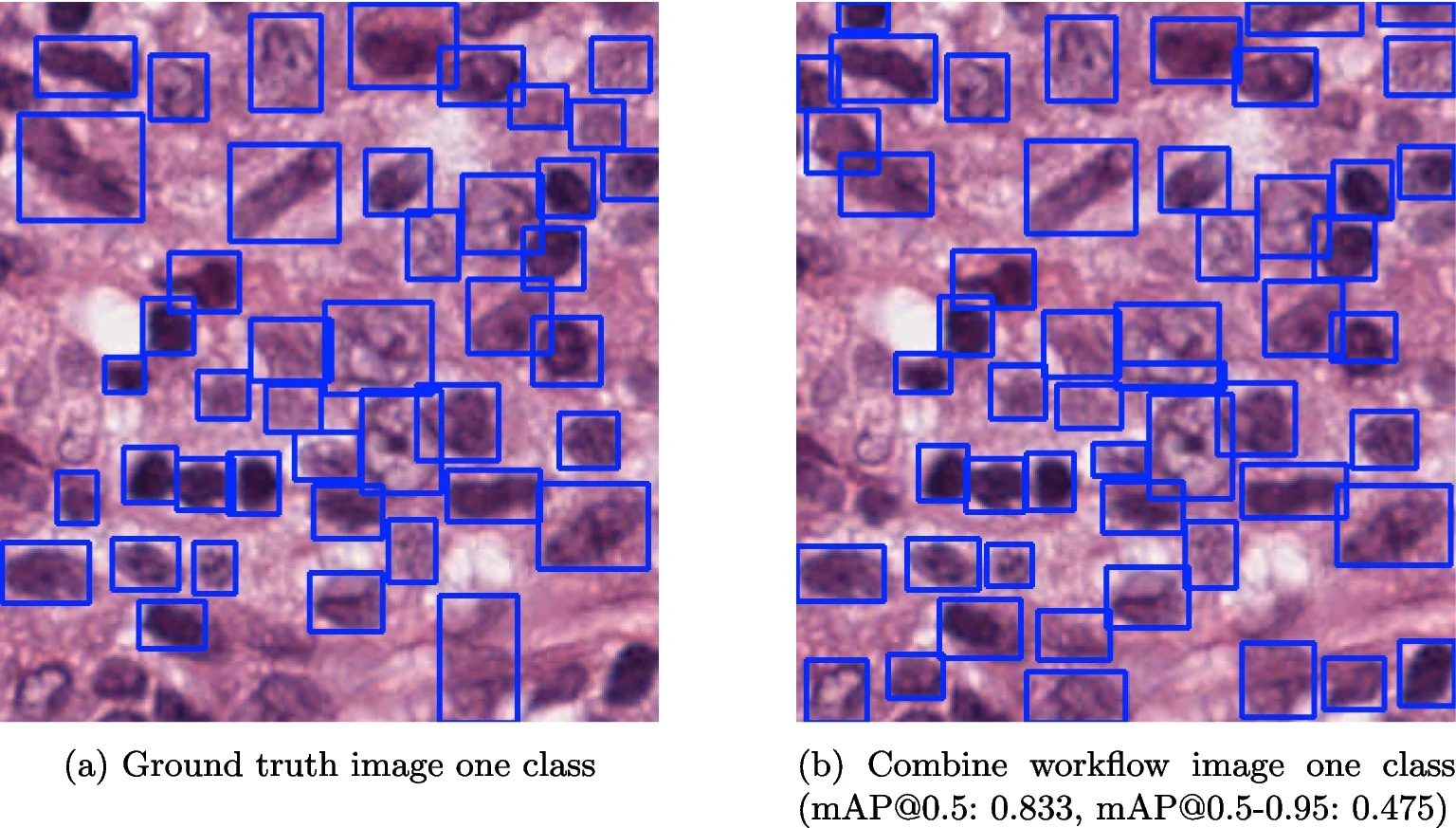
Illustration of the impact of classification on detection performance. When all detected objects are treated as belonging to a single class, the mAP@0.5–0.95 increases significantly, demonstrating that classification errors are a major limiting factor in the overall performance

This finding indicates that the models were effective in detecting the presence and location of objects, but exhibited notable limitations in correctly classifying them into their respective categories. To illustrate the impact of classification errors on the mAP score, Fig. 8a shows a hypothetical scenario in which all objects are considered to belong to the same class. In this case, the mAP@0.50-0.95 increases substantially—from 0.108 to 0.475—demonstrating that improved classification performance would significantly enhance the overall detection quality. The improvement is even more pronounced in the mAP@0.50 metric, which rises from 0.185 to 0.833 under the same condition, highlighting the exceptional effect of the classification issues on the evaluation metrics.

To improve the models in future works, we analysed the mean average precision (mAP@0.5–0.95) obtained for each class. The *tumor* class achieved an mAP of 0.390 with a standard deviation of 0.31, while the *sTIL* class reached 0.310 (± 0.33). The lowest performance corresponded to the *nonTIL* class, with an mAP of 0.254 (± 0.29), probably due to its visual similarity to the *sTIL* class, which often leads the model to confuse the two. Future research will therefore focus on developing strategies to reduce this misclassification between visually similar classes.

Further analysis reveals that the majority of classification errors occur between the nonTIL and sTIL classes, which exhibit strong visual similarity in histopathological images. This suggests that the classification challenge is largely influenced by the dataset characteristics rather than solely by model limitations. Moreover, the proposed fusion strategy primarily improves localization performance by increasing the number of correctly detected nuclei. However, it does not significantly alter the classification behavior of the detector, as class predictions are inherited from the underlying detection models. Therefore, while the combined workflow enhances detection robustness, classification accuracy remains an open challenge for future work.

### Computational cost analysis

Although the proposed combined workflow improves detection performance, it introduces additional computational overhead due to the dual inference process. Specifically, the need to process both the full image and multiple patches increases inference time compared to using a single workflow. However, this overhead remains manageable due to the efficiency of the YOLO V8 architecture, which enables fast inference even when applied multiple times. In practice, the increase in computational cost is offset by the gain in detection accuracy, particularly in challenging scenarios involving small or densely packed nuclei. Furthermore, the proposed framework can be parallelized, as patch-based inference can be executed concurrently, reducing overall processing time in multi-GPU or distributed environments. This makes the approach suitable for practical applications where both accuracy and efficiency are required.

## Conclusions

The experiments clearly show that combining detections from full-image and sliding-window pipelines substantially enhances detection accuracy without increasing model complexity. In particular, the YOLO V8-S model, when integrated into the combined workflow, achieves state-of-the-art performance while maintaining a lightweight architecture with fewer than ten million parameters. This makes it especially well-suited for practical deployment in computationally limited environments, such as point-of-care pathology stations. The weighted fusion mechanism proves effective in recovering true positives often missed by either approach in isolation, particularly under stringent confidence thresholds.

Overall, the method demonstrates reliable localization performance. The main limitation lies in the classification of morphologically similar nuclear types, which reduces overall mAP despite high recall. These results confirm the effectiveness of the proposed fusion strategy and its potential to improve detection robustness in histopathological tasks.

Although further refinements, particularly in class discrimination, could lead to additional gains, the current approach already represents a significant advance toward efficient and deployable models for digital pathology. Enhancements such as adaptive fusion or improved classification modules may be explored in future work, but the approach presented here provides a promising and practically viable foundation for future developments.

## Data Availability

The datasets utilized in this study are publicly accessible at https://sites.google.com/view/nucls/home. Additionally, the corresponding author can provide the data upon reasonable request.
